# Combined Atrial Functional Mitral and Tricuspid Regurgitation in Atrial Fibrillation: Prevalence, Associated Factors, and Three-Dimensional Valve Remodeling

**DOI:** 10.3390/jcm15135198

**Published:** 2026-07-02

**Authors:** Andrei-Alexandru Nour, Diana-Ruxandra Hădăreanu, Despina-Manuela Toader, Călin-Dinu Hădăreanu, Maria-Livia Iovănescu, Anca Mihu-Marinescu, Georgică-Costinel Târtea, Ionuț Donoiu, Edme-Roxana Mustafa, Oana Munteanu-Mirea, Răzvan-Ilie Radu, Octavian Istrătoaie, Cristina Florescu

**Affiliations:** 1 Doctoral School, University of Medicine and Pharmacy of Craiova, 2 Petru Rares St., 200349 Craiova, Romania; 2 Department of Cardiology, Filantropia Clinical Hospital, 28 Sararilor St., 200516 Craiova, Romaniacristina.t.florescu@umfcv.ro (C.F.); 3 Department of Cardiology, University of Medicine and Pharmacy of Craiova, 2 Petru Rares St., 200349 Craiova, Romaniaionut.donoiu@umfcv.ro (I.D.); roxana.mustafa@umfcv.ro (E.-R.M.); octavian.istratoaie@umfcv.ro (O.I.); 4 Department of Cardiology, Clinical Emergency County Hospital of Craiova, 1 Tabaci St., 200642 Craiova, Romania; 5 Department of Cardiovascular Surgery, Clinical Emergency County Hospital of Craiova, 1 Tabaci St., 200642 Craiova, Romania; 6 Department of Cardiology, Carol Davila University of Medicine and Pharmacy, 8 Eroii Sanitari Bld., 050474 Bucharest, Romania; 7 Department of Interventional Cardiology, Prof. Dr. C. C. Iliescu Emergency Institute for Cardiovascular Diseases, 258 Fundeni St., 022328 Bucharest, Romania

**Keywords:** atrial fibrillation, atrial functional regurgitation, mitral regurgitation, tricuspid regurgitation, three-dimensional echocardiography, transoesophageal echocardiography

## Abstract

**Background/Objectives**: Atrial fibrillation (AF) may cause functional mitral regurgitation (MR) and tricuspid regurgitation (TR) through atrial remodeling and annular dilation. However, the prevalence and structural characteristics of combined MR/TR in AF are not well defined. We aimed to determine the prevalence, clinical profile, and factors associated with combined clinically significant MR and TR in AF patients. **Methods**: In this prospective observational study (REMO-FIB), 175 consecutive AF patients underwent comprehensive transesophageal echocardiography with three-dimensional mitral valve analysis. After excluding organic MR and significant aortic valve disease, 125 patients were analyzed. Patients were classified into four groups according to the presence of moderate/severe MR and/or TR. Multivariable logistic regression evaluated factors associated with the combined phenotype. **Results**: Among 125 patients, 53 (42.4%) had no significant MR/TR, 33 (26.4%) had isolated MR, 11 (8.8%) had isolated TR, and 28 (22.4%) had combined MR/TR. Compared with patients without regurgitation, those with combined MR/TR had higher symptom burden (EHRA class, *p* = 0.036), more heart failure (92.9% vs. 67.9%, *p* = 0.048), larger left (47.0 vs. 42.0 mm, *p* = 0.002) and right atria (42.0 vs. 38.0 mm, *p* < 0.001), higher pulmonary artery pressure (40.0 vs. 28.0 mmHg, *p* = 0.004), and lower left ventricular ejection fraction (47.5% vs. 55.0%, *p* = 0.006). Three-dimensional analysis showed larger mitral annular perimeter (129.0 vs. 121.0 mm, *p* = 0.009), greater annular area (12.7 vs. 11.1 cm^2^, *p* = 0.014), longer anterior leaflet length (26.5 vs. 24.0 mm, *p* < 0.001), and greater tenting area (2.1 vs. 1.4 cm^2^, *p* = 0.002). Factors independently associated with the combined phenotype were female sex (OR 4.60, *p* = 0.015), lower ejection fraction (OR 0.47 per SD, *p* = 0.005), and larger right atrial diameter (OR 1.85 per SD, *p* = 0.037). Model discrimination was good (AUC 0.81). **Conclusions**: Combined moderate/severe MR and TR affects over one-fifth of AF patients without organic valve disease and is associated with advanced biatrial remodeling, adverse symptoms, and heart failure. Comprehensive assessment of both atrioventricular valves should be considered in AF.

## 1. Introduction

Atrial fibrillation (AF) is the most common sustained cardiac arrhythmia, affecting over 37 million individuals worldwide, with prevalence projected to increase substantially over the coming decades. Beyond its well-established associations with stroke and heart failure, AF is increasingly recognized as a driver of functional valvular heart disease through progressive atrial remodeling and annular dilation [1,2,3,4]. This distinct form of valvular dysfunction, termed “atrial functional regurgitation”, differs fundamentally from the classical ventricular functional regurgitation associated with left ventricular (LV) dilation and dysfunction, and may require different therapeutic approaches [1,2].

Atrial functional mitral regurgitation (AFMR) occurs in 7–28% of patients with AF, depending on arrhythmia duration, and results from left atrial (LA) dilation, causing mitral annular enlargement through flattening of the normal saddle-shaped annular geometry [2,3]. Loss of atriogenic annular contraction in AF further exacerbates regurgitation by delaying leaflet coaptation until systole [3]. Similarly, atrial functional tricuspid regurgitation (AFTR) develops through right atrial (RA) dilation and tricuspid annular enlargement, predominantly affecting the more pliable portion of the annulus along the right ventricular (RV) free wall [1,3]. Recent population-based studies have demonstrated that approximately one-third of patients with AF develop moderate or more severe tricuspid regurgitation (TR) over time, and this progression is associated with a more than two-fold increased mortality risk [5].

While mitral regurgitation (MR) and TR in AF have traditionally been studied as separate entities, emerging evidence suggests that these conditions frequently coexist, reflecting a shared pathophysiological substrate of biatrial remodeling [1,3]. Data from the ESC-HFA Heart Failure Long-Term Registry demonstrated that combined moderate or severe MR and TR was present in 11% of heart failure patients and was associated with the highest rates of adverse outcomes compared with isolated or absent valvular disease [6]. Analysis from the COAPT trial similarly showed that patients with secondary MR who also have moderate or more severe TR exhibit more severe clinical and echocardiographic characteristics and worse clinical outcomes [7].

Despite these observations, the specific phenotype of combined AFMR and AFTR in patients with AF remains incompletely characterized. The prevalence of this combined phenotype in AF populations without organic valve disease, its clinical correlates, the structural determinants that distinguish it from isolated valvular involvement, and the three-dimensional (3D) valvular changes underlying this condition have not been systematically evaluated.

The present study, conducted within the REMO-FIB (Remodeling in Atrial Fibrillation) project, was designed to address these knowledge gaps. We sought to (1) determine the prevalence of combined moderate or severe MR/TR in a prospective cohort of patients with persistent AF undergoing transesophageal echocardiography (TEE) before electrical cardioversion; (2) characterize the clinical profile, symptom burden, and therapeutic patterns across distinct valvular phenotypes; (3) define the echocardiographic features that distinguish patients with combined valvular disease from those with isolated or absent regurgitation; (4) explore the 3D mitral valve changes associated with the combined phenotype; and (5) identify factors independently associated with combined atrioventricular valve regurgitation. We hypothesized that the combined valvular phenotype would represent a distinct clinical and structural entity characterized by advanced biatrial remodeling and specific determinants that could inform patient stratification and integrated clinical assessment.

## 2. Materials and Methods

### 2.1. Study Design and Population

This was a prospective, observational study conducted within the REMO-FIB project, designed to characterize the overlap between clinically significant MR/TR in patients with persistent AF undergoing TEE for LA thrombi exclusion before electrical cardioversion, and to define the associated clinical and structural phenotype. A total of 175 consecutive patients with AF were prospectively enrolled in the overall database. For the purpose of the present analysis, patients were initially classified according to the etiology of MR as atrial functional, ventricular functional, or organic. In the overall cohort, AFMR represented the predominant mechanism, while a smaller proportion of patients exhibited ventricular functional MR and a minority had organic mitral valve disease.

**Figure 1:**
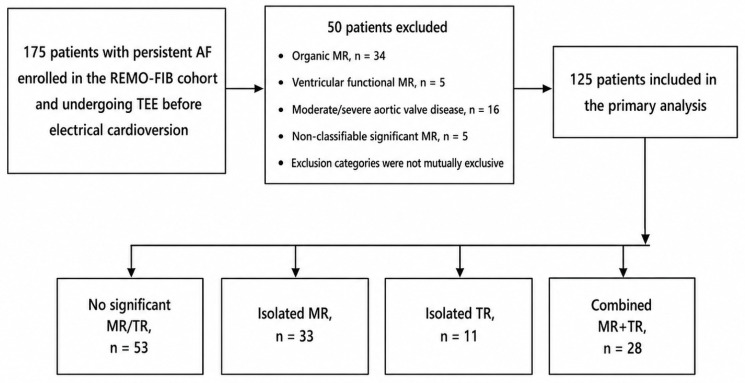
Patient selection flowchart.

To preserve the mechanistic coherence of the analysis and to avoid confounding by primary structural valve disease, the primary manuscript cohort was restricted to patients without organic MR and without moderate or severe aortic valvular disease. In addition, patients with significant MR in whom the mechanism could not be reliably classified were excluded from the primary analysis. After these exclusions, a total of 125 patients were included in the primary study population. Patient selection flowchart is presented in Figure 1.

### 2.2. Echocardiographic Assessment

All patients underwent comprehensive clinical evaluation and TEE. Standard echocardiographic assessment included LV ejection fraction (LVEF), LV dimensions, LA diameter, RV size, RA dimensions, tricuspid annular plane systolic excursion (TAPSE), systolic pulmonary artery pressure (sPAP), and TR maximal velocity [8,9,10]. MR and TR were graded according to standard multiparametric criteria and were dichotomized as non-significant when graded as none or mild and as clinically significant when graded as moderate or severe [11]. For MR, moderate regurgitation was defined by PISA-derived effective regurgitant orifice area (EROA) 20–39 mm^2^, regurgitant volume (RVol) 30–59 mL, and vena contracta (VC) width 3–6.9 mm, while severe MR was defined by EROA ≥ 40 mm^2^, regurgitant volume ≥ 60 mL, and vena contract width ≥ 7 mm. For TR, moderate regurgitation was defined by EROA 20–39 mm^2^, RVol 30–44 mL, and VC width 3–6.9 mm, while severe TR was defined by EROA ≥ 40 mm^2^, RVol ≥ 45 mL, and VC width ≥ 7 mm [11].

Three-dimensional mitral valve analysis was performed in all patients using TEE [12]. The analysis focused on parameters reflecting annular remodeling and leaflet adaptation, including annular perimeter, annular area, anteroposterior diameter, commissural diameter, annular height, anterior leaflet length, posterior leaflet length, tenting height, and tenting area. These parameters were chosen to provide a mechanistic link between atrial remodeling and valvular dysfunction. Based on the presence or absence of clinically significant MR and TR, patients were categorized into four phenotypic groups: those without significant regurgitation of either valve, those with isolated moderate or severe MR, those with isolated moderate or severe TR, and those with combined moderate or severe regurgitation affecting both valves.

All 3D mitral valve analyses were performed by the same experienced echocardiographer (D.-R.H.). Intraobserver and interobserver reproducibility of 3D mitral valve measurements was assessed in a randomly selected subset of 20 patients. For intraobserver variability, the same observer (D.-R.H.) repeated the measurements, blinded to the initial results. For interobserver variability, a second independent observer (M.-L.I.) repeated the measurements using the same 3D datasets, blinded to the first observer’s measurements. Reproducibility was quantified using intraclass correlation coefficients (ICCs).

### 2.3. Statistical Analysis

Continuous variables were assessed for distribution normality using visual inspection and the Shapiro–Wilk test. Normally distributed variables are presented as mean ± standard deviation and were compared using one-way analysis of variance. Non-normally distributed variables are presented as median [interquartile range] and were compared using the Kruskal–Wallis test. Categorical variables are presented as counts (percentages) and were compared using the chi-square test or Fisher’s exact test when appropriate.

To identify factors associated with the combined MR + TR phenotype, univariable logistic regression analyses were first performed. Variables considered clinically relevant and/or significant in univariable analysis were entered into a multivariable logistic regression model. Age and sex were included as demographic covariates, LVEF as a marker of ventricular function, LA diameter as a marker of left-sided atrial remodeling, and RA diameter as a marker of right-sided atrial remodeling. Continuous variables included in regression models were standardized per 1 standard deviation increment. No formal sample-size was performed, as this was an exploratory analysis of a prospectively enrolled cohort.

Model discrimination was evaluated using the area under the receiver-operating characteristic curve (AUC) with 95% confidence intervals. Calibration was assessed using the Brier score, calibration intercept, and calibration slope. Internal validation was performed using bootstrap resampling with 200 repetitions. Optimism-corrected discrimination was estimated by subtracting the mean bootstrap optimism from the apparent AUC. Model calibration optimism was similarly estimated using the calibration slope. Multicollinearity was evaluated using variance inflation factors. As a sensitivity analysis, penalized logistic regression with Firth correction was performed to reduce potential small-sample bias. Missing data were handled using complete-case analysis. A two-sided *p*-value < 0.05 was considered statistically significant. All analyses were performed using R version 4.5.3 for Mac (R Foundation for Statistical Computing, Vienna, Austria).

## 3. Results

Among the 175 patients included in the overall persistent AF cohort, clinically significant MR was present in 100 patients (57.1%), while clinically significant TR was present in 70 patients (40.0%). The combined phenotype of moderate or severe MR + TR was identified in 52 patients, representing 29.7% of the total cohort. With respect to MR mechanism, AFMR represented the dominant etiology, whereas ventricular functional MR accounted for a smaller subset of cases. Organic mitral valve disease was present in a minority of patients and was excluded from the primary mechanistic analysis.

After exclusion of patients with organic MR, moderate or severe aortic valvular disease, and cases with non-classifiable MR, the primary analysis included 125 patients. Within this cohort, 53 patients (42.4%) had neither clinically significant MR nor TR, 33 patients (26.4%) had isolated moderate or severe MR, 11 patients (8.8%) had isolated moderate or severe TR, and 28 patients (22.4%) exhibited the combined phenotype of moderate or severe regurgitation affecting both valves.

### 3.1. Clinical Profile Across Phenotypes

Baseline clinical characteristics are presented in Table 1.

#### 3.1.1. Demographic Characteristics

The median age of the overall cohort was 68.0 years (IQR 63.0–72.0), with a trend toward older age in patients with the combined phenotype (median 69.0 years, IQR 65.0–74.0) compared with those without significant regurgitation (median 65.0 years, IQR 61.0–69.0), although this difference did not reach statistical significance (*p* = 0.143). Female sex was more prevalent in the combined phenotype (53.6%) compared with the group without significant regurgitation (27.5%), with a borderline significant trend across groups (*p* = 0.087).

#### 3.1.2. Symptom Burden and Comorbidities

Symptom burden differed significantly across phenotypic groups (*p* = 0.036), with patients exhibiting isolated TR (median EHRA class 3.0, IQR 3.0–3.8) and those with the combined phenotype (median EHRA class 3.0, IQR 2.0–3.0) presenting with higher EHRA functional class compared with patients without significant regurgitation (median EHRA class 2.0, IQR 1.2–3.0) or isolated MR (median EHRA class 2.0, IQR 1.0–3.0). This finding suggests that right-sided involvement contributes significantly to symptom severity in this population. AF duration did not differ significantly across phenotypes (*p* = 0.959).

The overall burden of traditional cardiovascular risk factors was relatively similar across groups for most variables. A history of heart failure was significantly more frequent in the combined phenotype, where it was present in 92.9% of patients, compared with 84.8% in isolated MR, 72.7% in isolated TR, and 67.9% in patients without significant valvular regurgitation (*p* = 0.048). Dyslipidemia varied significantly across groups (*p* < 0.001), largely due to a markedly lower prevalence in the isolated TR subgroup (45.5%) compared with other groups (89.3–93.9%).

The therapeutic profile reflected the increasing complexity of the disease phenotype. Beta-blocker use was significantly different across groups (*p* = 0.029). SGLT2 inhibitor use increased progressively across phenotypes (*p* = 0.008), from 10.2% in patients without significant regurgitation to 42.9% in the combined phenotype. Loop diuretic use showed the most pronounced gradient across groups (*p* = 0.001), increasing from 27.1% in patients without significant regurgitation to 71.4% in the combined phenotype.

### 3.2. Echocardiographic Phenotype

Echocardiographic characteristics are summarized in Table 2.

#### 3.2.1. Left Ventricular and Left Atrial Parameters

LV systolic function, as assessed by LVEF, declined progressively across groups (*p* = 0.006). Patients without significant regurgitation had the highest median EF (55.0%, IQR 50.0–55.0%), followed by isolated MR (52.5%, IQR 48.0–55.0%), isolated TR (50.0%, IQR 50.0–53.5%), and the combined phenotype (47.5%, IQR 42.2–52.2%). Although all median values remained within the preserved or mildly reduced range, the progressive decline across phenotypes suggests that subtle ventricular-atrial coupling abnormalities may contribute to the development of combined valvular disease.

LA size increased significantly across phenotypes (*p* = 0.002), supporting the central role of atrial remodeling in the pathophysiology of valvular dysfunction in AF. Patients without significant regurgitation had the smallest LA diameter (median 42.0 mm, IQR 39.5–45.0), followed by isolated MR (median 46.0 mm, IQR 42.0–49.0), isolated TR (median 46.0 mm, IQR 45.5–47.0), and the combined phenotype (median 47.0 mm, IQR 42.2–53.0).

#### 3.2.2. Right-Sided Parameters

Right-sided parameters showed an even more pronounced pattern of structural abnormalities across phenotypes. SPAP increased markedly across groups (*p* = 0.004), from a median of 28.0 mmHg (IQR 24.5–30.0) in patients without significant regurgitation to 40.0 mmHg (IQR 33.5–45.8) in the combined phenotype.

RA diameter was strongly associated with valvular phenotype (*p* = 0.001), with the largest dimensions observed in patients with combined MR + TR (median 42.0 mm, IQR 41.0–45.8) and isolated TR (median 41.5 mm, IQR 40.0–49.5), compared with isolated MR (median 38.0 mm, IQR 35.0–41.0) and patients without significant regurgitation (median 38.0 mm, IQR 36.0–40.5).

### 3.3. Three-Dimensional Mitral Valve Analysis

Three-dimensional mitral valve parameters are presented in Table 3.

Reproducibility of 3D mitral valve measurements was good to excellent. Intraobserver ICCs ranged from 0.939 to 0.990, while interobserver ICCs ranged from 0.920 to 0.989. The intraobserver/interobserver ICCs were 0.979/0.966 for annular perimeter and 0.960/0.949 for tenting area.

#### 3.3.1. Annular Remodeling and Geometry

Patients with combined MR + TR exhibited larger annular dimensions compared with patients without significant regurgitation. Annular perimeter was significantly increased (129.0 mm [IQR 120.8–134.2] vs. 121.0 mm [IQR 114.0–128.0], *p* = 0.009), as was annular area (12.7 cm^2^ [IQR 11.2–13.9] vs. 11.1 cm^2^ [IQR 10.0–12.7], *p* = 0.014). Anteroposterior diameter was also significantly larger in the combined phenotype (38.0 mm [IQR 36.0–41.2] vs. 36.0 mm [IQR 32.0–39.0], *p* = 0.034).

Notably, patients with isolated MR demonstrated the most pronounced annular dilation (perimeter 130.0 mm, area 12.8 cm^2^), while patients with isolated TR showed intermediate values. This pattern suggests that annular dilation is the primary driver of isolated MR, whereas the combined phenotype involves additional mechanisms.

Annular height differed significantly across groups (*p* = 0.019), with the lowest values observed in isolated TR (4.6 mm [IQR 3.1–7.4]), indicating annular flattening. In contrast, patients with isolated MR maintained relatively preserved annular height (8.1 mm [IQR 6.3–9.1]), suggesting preservation of the saddle shape despite dilation. The combined phenotype showed intermediate annular height (7.8 mm [IQR 6.8–8.6]).

#### 3.3.2. Leaflet Adaptation and Tenting Parameters

Anterior leaflet length was significantly greater in patients with valvular regurgitation compared with those without (*p* = 0.001). Patients with isolated MR had the longest anterior leaflets (28.0 mm [IQR 27.0–29.0]), followed by the combined phenotype (26.5 mm [IQR 24.0–31.0]), isolated TR (25.0 mm [IQR 21.5–27.0]), and patients without significant regurgitation (24.0 mm [IQR 21.0–26.0]). This finding suggests compensatory leaflet elongation in response to annular dilation. However, the presence of significant regurgitation despite increased leaflet length indicates that this adaptation was insufficient to maintain coaptation.

Tenting height (*p* = 0.005) and tenting area (*p* = 0.002) differed significantly across groups. The combined phenotype exhibited the greatest tenting abnormalities (tenting height 7.0 mm [IQR 6.0–9.0]; tenting area 2.1 cm^2^ [IQR 1.5–2.6]), while isolated TR showed the lowest tenting values (tenting height 3.0 mm [IQR 3.0–6.0]; tenting area 1.2 cm^2^ [IQR 0.8–1.7]). This pattern suggests that the combined phenotype involves not only annular dilation but also altered leaflet geometry and subvalvular mechanics, even in the context of predominantly atrial functional disease.

### 3.4. Factors Associated with the Combined Phenotype

Logistic regression analysis was performed to identify factors associated with the presence of combined moderate or severe MR + TR, as shown in Table 4.

#### 3.4.1. Univariable Analysis

In univariable analysis, several echocardiographic parameters were significantly associated with the combined phenotype. Lower LVEF was associated with increased odds of the combined phenotype (OR 0.50 per SD; 95% CI 0.33–0.77; *p* = 0.002), indicating that for each standard deviation decrease in EF, the odds of having the combined phenotype approximately doubled. Larger LA diameter was associated with increased odds of the combined phenotype (OR 1.76 per SD; 95% CI 1.11–2.80; *p* = 0.017), consistent with the central role of LA remodeling in the pathophysiology of atrial functional valvular disease. Larger RA diameter showed a particularly strong association with the combined phenotype (OR 1.93 per SD; 95% CI 1.22–3.06; *p* = 0.005), highlighting the importance of right-sided remodeling in the development of combined valvular disease.

Among clinical variables, the use of SGLT2 inhibitors (OR 3.61; 95% CI 1.44–9.08; *p* = 0.006) and loop diuretics (OR 4.26; 95% CI 1.69–10.73; *p* = 0.002) was associated with the combined phenotype. These associations likely reflect heart failure severity rather than causality, as patients with more advanced valvular disease and heart failure are more likely to receive these therapies.

#### 3.4.2. Multivariable Analysis

In the multivariable model, which included age, sex, LVEF, LA diameter, and RA diameter, three variables remained independently associated with the combined phenotype.

Female sex was independently associated with the combined phenotype (OR 4.60; 95% CI 1.35–15.69; *p* = 0.015), indicating that women had more than four-fold higher odds of having the combined phenotype compared with men, after adjustment for other variables. This finding is consistent with recent literature demonstrating sex differences in the development and progression of AFMR. Lower LVEF remained independently associated with the combined phenotype (OR 0.47 per SD; 95% CI 0.27–0.79; *p* = 0.005), confirming that subtle LV dysfunction contributes to the development of combined valvular disease even after adjustment for atrial dimensions. Finally, larger RA diameter also remained independently associated with the combined phenotype (OR 1.85 per SD; 95% CI 1.04–3.30; *p* = 0.037), confirming the importance of right-sided remodeling in the development of combined valvular disease.

LA diameter showed a borderline association with the combined phenotype (OR 1.78 per SD; 95% CI 0.99–3.21; *p* = 0.054). The attenuation of this association in the multivariable model may reflect collinearity with RA diameter, as both atria tend to dilate together in the context of AF and heart failure. Age was not independently associated with the combined phenotype in the multivariable model (OR 1.18 per SD; 95% CI 0.65–2.12; *p* = 0.584), suggesting that the effects of age on valvular disease are mediated through other variables in the model.

**Figure 2:**
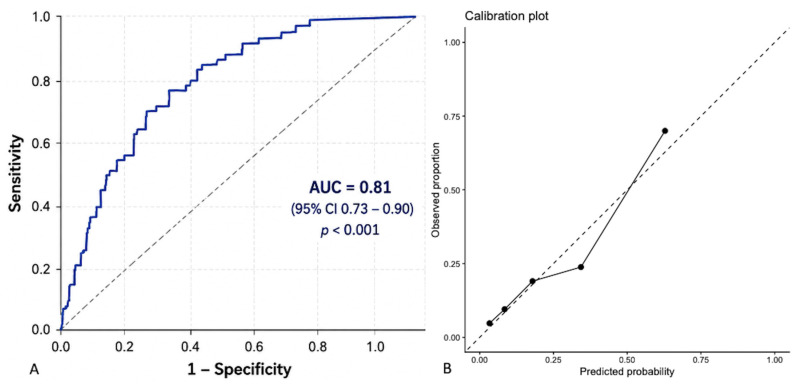
Model performance of the exploratory multivariable model for identifying the combined phenotype. (**A**) Receiver-operator characteristics curve showing an apparent AUC of 0.81. (**B**) Calibration plot comparing predicted probabilities with observed proportions across risk groups. The dashed line represents perfect calibration. AUC, area under the curve; CI, confidence interval.

Model performance is shown in Figure 2, including the receiver-operating characteristics curve (Figure 2A) and calibration plot (Figure 2B). The exploratory multivariable model showed good apparent discrimination for identifying the combined MR + TR phenotype, with an area under the receiver-operating characteristic (AUC) curve of 0.81 (95% CI 0.71–0.91). Predictive accuracy was supported by a Brier score of 0.133. Apparent calibration showed a calibration slope of 1.00 and a calibration intercept near zero; however, internal validation using 200 bootstrap resamples showed a mean AUC optimism of 0.040, resulting in an optimism-corrected AUC of 0.77. The optimism-corrected calibration slope was 0.79, indicating some degree of overfitting, consistent with the limited number of events. Multicollinearity among variables was low, with all variance inflation factors below 1.4: age, 1.10; female sex, 1.30; LVEF, 1.12; LA diameter, 1.18; and RA diameter, 1.39. In sensitivity analysis using penalized logistic regression with Firth correction, the direction and magnitude of associations remained consistent, confirming robustness of the primary findings. Female sex, lower LVEF, and larger RA diameter remained independently associated with the combined phenotype.

Taken together, the data identify a distinct clinical and structural phenotype characterized by the coexistence of moderate or severe MR/TR in patients with AF. This phenotype is associated with greater symptom burden (higher EHRA functional class), higher prevalence of heart failure (92.9% vs. 67.9%), and more intensive medical therapy, including greater use of SGLT2 inhibitors and loop diuretics. Patients also demonstrated larger LA size (median 47.0 mm vs. 42.0 mm) and RA size (median 42.0 mm vs. 38.0 mm), higher pulmonary pressures (median sPAP 40.0 mmHg vs. 28.0 mmHg), and subtle impairment of LV systolic function (median LVEF 47.5% vs. 55.0%). Structural assessment further showed mitral annular dilation with insufficient leaflet adaptation and increased tenting area, suggesting altered leaflet geometry.

The integration of conventional echocardiography and 3D mitral valve analysis suggests that this phenotype reflects a global remodeling process involving both atria and both atrioventricular valves, rather than isolated disease of a single valve. This integrated perspective has important implications for patient stratification, therapeutic decision-making, and the design of future interventional studies.

## 4. Discussion

The present study provides a comprehensive characterization of the overlap between clinically significant MR/TR in patients with AF, identifying a distinct combined valvular disease phenotype associated with advanced biatrial remodeling, increased symptom burden, and specific structural features. The main findings include: (1) the combined phenotype of moderate or severe AFMR + AFTR is present in approximately one-fifth of AF patients without organic valvular disease; (2) female sex, RA dilation, and subtle LV dysfunction are independently associated with this phenotype; (3) patients with combined valvular disease exhibit a progressive structural continuum characterized by biventricular and biatrial remodeling; and (4) 3D analysis reveals mitral annular remodeling and insufficient leaflet adaptation as key mechanisms of valvular dysfunction. Importantly, the exploratory multivariable model demonstrated favorable internal performance, with good discrimination and excellent calibration. These findings suggest that a limited set of routinely available clinical and echocardiographic variables may permit practical identification of patients at risk for the combined phenotype. Although external validation is required, the present results support the feasibility of phenotype-oriented risk stratification in AF-associated valvular disease.

### 4.1. Prevalence and Clinical Significance of Combined Valvular Disease in Atrial Fibrillation

The 22.4% prevalence of combined moderate or severe MR + TR in our primary cohort, and 29.7% in the overall AF cohort, represents a substantial clinical burden that has been underappreciated in prior literature. These prevalence rates are higher than the 11% reported in the ESC-HFA Heart Failure Long-Term Registry [6], which included a broader heart failure population with varying EFs. The higher prevalence in our cohort likely reflects the specific selection of patients with AF undergoing TEE, which may identify more advanced disease and provides more accurate assessment of regurgitation severity.

Recent population-based studies have demonstrated that approximately one-third of patients with AF develop moderate or more severe TR over time [5]. In an observational study of patients with newly diagnosed AF in the absence of structural heart disease, 33.6% developed moderate or greater TR during a follow-up period of 13 years, with older patients, women, and those with persistent or permanent AF being more likely to develop TR [13]. Similarly, AFMR occurs in 7–28% of patients with AF [14], depending on arrhythmia duration, with significant MR more frequently seen in patients with AF duration exceeding 10 years (27% vs. 4% in patients with shorter duration) [15].

Our findings extend these observations by demonstrating that when both valves are affected, the clinical and structural burden is substantially greater than with isolated involvement of either valve. The observation that patients with the combined phenotype exhibit significantly greater symptom burden (higher EHRA class) and a higher prevalence of heart failure (92.9% vs. 67.9% in the group without significant regurgitation) underscores the clinical impact of this entity. Data from the ESC-HFA Registry [6] demonstrated that all-cause death, cardiovascular death, heart failure hospitalization, and combined outcomes occurred more frequently in combined MR + TR, isolated TR, and isolated MR versus no regurgitation, with the highest incident rates observed in isolated TR and combined MR + TR.

Analysis from the COAPT trial [7] similarly showed that patients with severe secondary MR who also have moderate or more severe TR have more severe clinical and echocardiographic characteristics and worse clinical outcomes compared with those with mild or less TR. At 2 years, the composite rate of death or heart failure hospitalization was significantly higher in patients with moderate or severe TR compared with mild or less TR treated with guideline-directed medical therapy alone (83.0% vs. 64.3%; hazard ratio 1.74; 95% CI 1.24–2.45; *p* = 0.001). Furthermore, Agricola et al. [16] demonstrated that moderate or more functional TR is independently associated with worse survival and a high incidence of heart failure episodes in patients with functional MR, with survival free of all-cause mortality at 6 years of 69%, 67%, 51%, and 40% for patients without, and with mild, moderate, and severe functional TR, respectively (*p* = 0.004).

### 4.2. Biatrial Remodeling as a Substrate for Combined Atrial Functional Regurgitation

Our study demonstrates that LA and RA dilation represent key structural determinants of the combined phenotype, with RA diameter remaining independently associated with the combined phenotype in multivariable analysis (OR 1.85; *p* = 0.037). This finding is consistent with recent literature highlighting the central role of RA remodeling in the development of AFTR [17,18,19,20].

The mechanism by which AF leads to valvular regurgitation involves several interconnected pathways [3]. In AFMR, LA dilation causes mitral annular dilation through flattening of the normal saddle-shaped annular geometry, increasing its projected area [2,3]. The fibrous composition of the mitral annulus precludes intrinsic motion; annular contraction is instead facilitated by the motion of adjacent atrial and ventricular musculature. During late diastole, the mitral annulus narrows because of contraction of the circumferential fibers (arising from Bachmann’s bundle) that surround the base of the LA (atriogenic annular contraction), whereas in systole, annular narrowing is facilitated by contraction of the superficial oblique fibers of the LV inlet (ventriculogenic annular contraction). Loss of the atriogenic component of annular contraction in AF increases MR by delaying the onset of leaflet coaptation until systole [3].

Similarly, for TR, RA dilation induces tricuspid annular dilation, predominantly affecting the more pliable and adipose tissue-rich portion of the annulus located along the RV free wall [17,19]. AFTR is characterized by normal-appearing leaflets that fail to coapt in the presence of marked annular and atrial dilatation, with minimal leaflet tethering or tenting and typically normal RV structure and function [17,21,22]. The association between tricuspid annular dilation and TR has been well established [18], with RA enlargement occurring before RV dilation, which occurs late and is associated with more severe TR [21].

Recent studies have demonstrated that the ratio of RA area to RV end-systolic area predicts progression to significant AFTR, suggesting that disproportionate atrial dilation relative to ventricular size creates a substrate for valvular regurgitation [17,23]. Harada et al. [23] demonstrated that RA dilation was common (78%) in patients with significant TR in heart failure with preserved EF, exceeding the prevalence of RV dilation (32%), and that RA dilation was strongly correlated with tricuspid annular diameter and TR vena contracta width (r = 0.67 and r = 0.70, both *p* < 0.0001). This observation is particularly relevant to our cohort, in which patients with the combined phenotype exhibited marked RA dilation (median diameter 42.0 mm) in the absence of severe RV dilation, supporting the predominantly atrial phenotype. Nonetheless, RA evaluation has important prognostic implications [24].

Importantly, Muraru et al. [17] recently proposed that AFTR accounts for 10–15% of clinically relevant TR and has better outcomes compared with the more prevalent ventricular phenotype, suggesting that patients with AFTR may benefit from more aggressive rhythm control and timely valve interventions. However, they also noted that a large proportion of patients present with mixed features in the late stages of disease, highlighting the importance of early identification and intervention [19].

### 4.3. Insufficient Leaflet Adaptation and Three-Dimensional Remodeling

Our 3D mitral valve analysis revealed that patients with the combined phenotype exhibit not only larger annular dimensions (increased perimeter and area) but also increased anterior leaflet length and greater tenting area. These findings suggest that while some leaflet adaptation occurs (increased length), it is insufficient to compensate for annular dilation, resulting in malcoaptation and regurgitation [2,25].

The concept of insufficient leaflet adaptation has been proposed as a key mechanism in AFMR [2]. Studies have demonstrated large disparities in MR burden despite similar amounts of mitral annular dilation, suggesting that adaptive leaflet growth varies among individuals [2,25]. Naser et al. [25] observed that after median 2.2 years of follow-up, the progression of mild-moderate/moderate to severe AFMR was uncommon, occurring in only 1.9% of patients every year, while regression occurred in 3.6% per year. This suggests that the balance between annular dilation and leaflet adaptation determines whether regurgitation progresses or regresses over time.

Factors influencing the adequacy of leaflet adaptation include age, sex, neurohormonal balance (particularly renin–angiotensin–aldosterone system activation), and the fibroblastic response to mechanical stress [25]. Sex hormones can modify the response of fibroblasts to the stress inflicted by changes in mitral annular dynamics and can be implicated in inadequate compensatory growth of the leaflets in the setting of LA enlargement. Furthermore, the composition of mitral and tricuspid annuli is different between sexes, with less/absent myocardium and less collagen matrix and elasticity, potentially contributing to the propensity toward annular dilation in women [25].

The increased tenting area observed in our cohort also indicates that altered leaflet geometry and subvalvular mechanics contribute to regurgitation severity even in the absence of significant ventricular remodeling. In particular, the greater tenting area in the combined MR + TR group, despite annular dimensions comparable to isolated MR, underscores that AFMR is not purely an annular disease but involves complex remodeling of the entire valvular apparatus. Recent studies using 3D computed tomography have demonstrated that decreased interpapillary muscle distance is strongly associated with improvements in AFMR after catheter ablation, suggesting that subvalvular geometry plays a role even in predominantly atrial disease [26]. Nonetheless, although organic MR and overt ventricular functional MR were excluded, the lower LVEF, higher pulmonary artery pressures, and greater heart failure burden observed in the combined MR + TR group raise the possibility of a mixed atrial-ventricular functional mechanism in some patients.

### 4.4. Sex Differences in Atrial Functional Valvular Disease

An important finding of our study is that female sex was independently associated with the combined phenotype in multivariable analysis (OR 4.60; 95% CI 1.35–15.69; *p* = 0.015). This observation is consistent with recent literature demonstrating that women have at least a two-fold higher risk of developing and progressing to severe AFMR compared with men [25,27].

Naser et al. [25] demonstrated that female sex was independently associated with progression of AFMR (HR 2.66; 95% CI 1.27–5.58; *p* = 0.01), while male sex was associated with regression. Similarly, in a study of incident AFMR, female sex was an independent risk factor in both AF and sinus rhythm populations [27]. For TR, observational studies have shown that older patients, women, and those with persistent or permanent AF were more likely to develop TR [19].

Several mechanisms have been proposed to explain this female predisposition. Women with AF exhibit a greater burden of atrial fibrosis compared with men, possibly due to presentation at a more advanced disease stage in the context of atypical symptoms or a different response to the inflammation underlying AF as a result of sex hormones [25]. Sex hormones may also modify the fibroblastic response to mechanical stress and influence compensatory leaflet growth. Furthermore, the composition of the mitral and tricuspid annuli differs between sexes, with less myocardium and less collagen matrix and elasticity in women, potentially contributing to greater propensity for annular dilation [25]. Recent data also suggest sex-specific patterns of right heart remodeling and adverse outcomes in secondary TR, supporting the concept that women may be particularly susceptible to advanced disease [28].

These sex differences have important clinical implications. Women with AF and valvular disease may benefit from earlier and more aggressive rhythm control strategies, closer echocardiographic surveillance, and potentially earlier consideration of interventional therapies. The independent association of female sex with the combined phenotype in our study supports the need for sex-specific approaches to the management of atrial functional valvular disease.

### 4.5. The Role of Subtle Left Ventricular Dysfunction

Although our cohort was restricted to patients with functional regurgitation (excluding organic valve disease), we observed that lower LVEF remained independently associated with combined phenotype (OR 0.47 per SD; *p* = 0.005), even though median values remained in the normal or mildly reduced range (47.5% in the combined group vs. 55.0% in the group without significant regurgitation).

This finding suggests that subtle LV systolic dysfunction, possibly related AF-induced cardiomyopathy or volume overload from valvular regurgitation, contributes to the development of the combined phenotype [3]. Impaired LV systolic function, not infrequently associated with AF, causes a reduction in left ventricular basal twist, which may potentially impair ventriculogenic annular contraction [3]. Additionally, LV dysfunction may contribute to increased LA pressures, exacerbating atrial remodeling and annular dilation.

Importantly, Flachskampf et al. [29] recently noted that because of the normal-sized and normally or nearly normally contracting LV in AFMR, the amount of regurgitation is typically not more than moderate or moderate-to-severe by standard criteria. However, they also emphasized that there is a large overlap of symptoms and signs between AFMR and heart failure with preserved EF, making it likely that the role of AFMR in patients with heart failure has been underestimated [29].

### 4.6. Therapeutic and Clinical Implications, and Future Directions

From a therapeutic perspective, emerging data suggest that rhythm control in AF may lead to reverse remodeling and reduction in valvular regurgitation severity [30]. Studies have demonstrated that maintenance of sinus rhythm after cardioversion or catheter ablation is associated with reduction in LA volume, RA area, tricuspid annular diameter, and regurgitation severity [18,30,31]. Importantly, AF ablation may be less successful in patients with chronic TR, underscoring the importance of early intervention [2].

In this context, our findings support phenotype-oriented patient stratification rather than direct therapeutic decision-making. The relatively high prevalence of combined MR + TR in patients with persistent AF (22.4% in the primary cohort) underscores the need for comprehensive echocardiographic assessment of both atrioventricular valves. The association of the combined phenotype with female sex, RA enlargement, lower LVEF, higher pulmonary artery pressures, greater symptom burden, and higher heart failure prevalence may help identify patients who require closer follow-up, optimization of rhythm-control and heart failure therapy, and multidisciplinary evaluation when clinically significant regurgitation persists.

For patients with advanced combined valvular disease who are not candidates for or have failed rhythm control strategies, transcatheter approaches targeting the mitral valve (edge-to-edge repair, annuloplasty) or the tricuspid valve may be considered. In patients being considered for transcatheter mitral valve interventions, structured echocardiographic screening remains essential, and recent data have supported relevant correlations between transthoracic and TEE assessment during transcatheter edge-to-edge repair evaluation [32]. However, the present study was observational and did not assess longitudinal clinical outcomes, procedural eligibility, intervention timing, or post-interventional outcomes. Therefore, our data should be interpreted as supporting integrated valve assessment and risk stratification in AF, while future longitudinal studies are needed to determine whether early rhythm-control strategies or valve-directed interventions can improve outcomes in patients with combined AFMR and AFTR.

### 4.7. Limitations

The study has several limitations that merit mention. First, the relatively small sample size, particularly for the isolated TR subgroup (n = 11), represents an important limitation affecting the detection of more subtle differences between groups. Moreover, the combined MR + TR group included 28 events; therefore, the events-per-variable ratio of the multivariable model was limited. Second, the cross-sectional nature of the analysis does not allow evaluation of valvular disease progression over time or the impact on long-term clinical outcomes. Third, although cases with organic valvular disease were excluded, the distinction between atrial and ventricular functional regurgitation may be difficult in cases with mixed features, particularly in advanced disease stages. Fourth, the study was conducted at a single center within a specific project (REMO-FIB) and included patients with persistent AF referred for TEE before electrical cardioversion. Therefore, the findings may not be generalizable to other AF populations. Fifth, volumetric parameters of atrial size were not used in the analysis, given the TEE assessment, and atrial remodeling was assessed using linear dimensions, which may misestimate atrial enlargement in patients with non-spherical atrial geometry, particularly in long-standing AF. Finally, 3D analysis was performed only for the mitral valve, since dedicated 3D tricuspid valve datasets were not available in all patients; future studies incorporating 3D tricuspid valve assessment would provide additional mechanistic insights.

## 5. Conclusions

Combined moderate or severe MR + TR represents a distinct phenotype in patients with AF, affecting approximately one-fifth of patients with functional atrial regurgitation and associated with greater symptom burden, heart failure prevalence, and advanced biatrial remodeling. Female sex, RA dilation, and lower LVEF were independently associated with the combined phenotype. Three-dimensional TEE demonstrated that mitral annular dilation and insufficient leaflet adaptation are key mechanisms underlying valvular dysfunction. These findings support the concept of progressive atrioventricular remodeling in AF and may help improve patient stratification and therapeutic decision-making.

## Figures and Tables

**Table 1:** Baseline Clinical Characteristics.

Variable	Overall (n = 125)	No MR/TR (n = 53)	Isolated MR (n = 33)	Isolated TR (n = 11)	Combined MR + TR (n = 28)	*p*-Value
Demographics						
Age, years	68.0 [63.0–72.0]	65.0 [61.0–69.0]	68.0 [63.0–72.0]	68.0 [63.0–72.5]	69.0 [65.0–74.0]	0.143
Female sex	50/123 (40.7%)	14/51 (27.5%)	16/33 (48.5%)	5/11 (45.5%)	15/28 (53.6%)	0.087
AF duration, months	3.0 [1.0–6.0]	3.0 [2.0–6.0]	2.0 [1.0–6.0]	3 [2.0–4.0]	3.0 [2.0–6.0]	0.959
EHRA class	2.0 [1.2–3.0]	2.0 [1.2–3.0]	2.0 [1.0–3.0]	3.0 [3.0–3.8]	3.0 [2.0–3.0]	0.036
CHA_2_DS_2_-VASc score	3.0 [2.0–4.0]	3.0 [2.0–3.5]	3.0 [2.0–4.0]	3.0 [2.0–3.5]	3.0 [2.0–4.0]	0.711
HAS-BLED score	1.0 [1.0–1.0]	1.0 [0.0–1.0]	1.0 [0.0–1.0]	1.0 [1.0–1.0]	1.0 [1.0–1.2]	0.233
Comorbidities						
Hypertension	107 (85.6%)	41 (77.4%)	31 (93.9%)	10 (90.9%)	25 (89.3%)	0.680
Diabetes mellitus	38 (30.4%)	14 (26.4%)	11 (33.3%)	4 (36.4%)	9 (32.1%)	0.946
Dyslipidemia	105 (84%)	44 (83%)	31 (93.9%)	5 (45.5%)	25 (89.3%)	0.001
Smoking	19 (15.2%)	10 (18.9%)	6 (18.2%)	1 (9.1%)	2 (7.1%)	0.410
Obesity	44 (35.2%)	18 (34%)	11 (33.3%)	3 (27.3%)	12 (42.9%)	0.792
Coronary artery disease	17 (13.6%)	6 (11.3%)	6 (18.2%)	1 (9.1%)	4 (14.3%)	0.844
Prior stroke/embolism	9 (7.2%)	4 (7.5%)	1 (3%)	2 (18.2%)	2 (7.1%)	0.421
COPD	3 (2.4%)	0 (0%)	2 (6.1%)	0 (0%)	1 (3.6%)	0.339
Thyroid disease	16 (12.8%)	4 (7.5%)	3 (9.1%)	3 (27.3%)	6 (21.4%)	0.130
Any heart failure	98 (78.4%)	36 (67.9%)	28 (84.8%)	8 (72.7%)	26 (92.9%)	0.048
CKD	41 (32.8%)	16 (30.2%)	12 (36.4%)	3 (27.3%)	10 (35.7%)	0.944
Medications						
Beta-blocker	99 (79.2%)	36 (67.9%)	31 (93.9%)	7 (63.6%)	25 (89.3%)	0.029
RAAS inhibitor	96 (76.8%)	35 (66%)	28 (84.8%)	9 (81.8%)	24 (85.7%)	0.359
Any antiarrhythmic	94 (75.2%)	41 (77.4%)	25 (75.8%)	8 (72.7%)	20 (71.4%)	0.943
Oral anticoagulation	115 (92%)	47 (88.7%)	30 (90.9%)	11 (100%)	27 (96.4%)	0.580
Antiplatelet therapy	10 (8%)	2 (3.8%)	7 (21.2%)	0 (0%)	1 (3.6%)	0.017
SGLT2 inhibitor	28 (22.4%)	5 (9.4%)	7 (21.2%)	4 (36.4%)	12 (42.9%)	0.008
Loop diuretic	54 (43.2%)	13 (24.5%)	14 (42.4%)	7 (63.6%)	20 (71.4%)	0.001
Cardioversion attempted	88 (70.4%)	37 (69.8%)	25 (75.8%)	10 (90.9%)	16 (57.1%)	0.132
Successful cardioversion	83 (94.3%)	35 (66%)	24 (72.7%)	9 (81.8%)	15 (53.6%)	0.787
Laboratory						
Creatinine, mg/dL	1.0 [0.8–1.2]	1.1 [0.8–1.2]	1.0 [0.8–1.2]	1.0 [0.8–1.0]	0.9 [0.8–1.3]	0.569
eGFR, mL/min/1.73 m^2^	68.0 [54.0–82.3]	69.1 [51.9–82.3]	68.0 [53.9–76.6]	71.6 [61.1–91.5]	65.3 [54.6–77.7]	0.623
NT-proBNP, pg/mL	857.5 [575.2–1517.5]	690.0 [304.0–1030.5]	857.5 [661.5–1445.5]	1187.2 [533.9–2820.5]	1284.0 [620.2–2262.0]	0.216

Data are presented as median [interquartile range] or n/N (%). AF = atrial fibrillation; CKD = chronic kidney disease; COPD = chronic obstructive pulmonary disease; eGFR = estimated glomerular filtration rate; MR = mitral regurgitation; NT-proBNP = N-terminal pro-B-type natriuretic peptide; RAAS = renin–angiotensin–aldosterone system; SGLT2 = sodium-glucose cotransporter 2; TR = tricuspid regurgitation.

**Table 2:** Echocardiographic Characteristics.

Variable	Overall (n = 125)	No MR/TR (n = 53)	Isolated MR (n = 33)	Isolated TR (n = 11)	Combined MR + TR (n = 28)	*p*-Value
Left Heart						
LVEF, %	50.0 [45.0–55.0]	55.0 [50.0–55.0]	52.5 [48.0–55.0]	50.0 [50.0–53.5]	47.5 [42.2–52.2]	0.006
LV end-diastolic diameter, mm	48.0 [45.0–52.2]	48.0 [45.0–50.0]	49.0 [45.0–53.0]	45.0 [42.0–49.5]	49.0 [46.0–55.8]	0.119
LA diameter, mm	45.0 [41.0–48.0]	42.0 [39.5–45.0]	46.0 [42.0–49.0]	46.0 [45.5–47.0]	47.0 [42.2–53.0]	0.002
Right Heart						
sPAP, mmHg	33.0 [27.0–44.5]	28.0 [24.5–30.0]	33.0 [23.0–44.0]	36.5 [34.0–51.8]	40.0 [33.5–45.8]	0.004
TR Vmax, m/s	2.7 [2.3–3.0]	2.3 [1.9–2.5]	2.7 [1.6–3.0]	2.8 [2.7–3.0]	2.8 [2.6–3.0]	0.008
RV basal diameter, mm	36.0 [33.0–38.2]	36.0 [32.0–37.0]	35.0 [33.0–38.0]	37.5 [36.2–41.5]	37.5 [34.0–39.8]	0.109
RA diameter, mm	40.0 [37.0–44.0]	38.0 [36.0–40.5]	38.0 [35.0–41.0]	41.5 [40.0–49.5]	42.0 [41.0–45.8]	0.001
TAPSE, mm	19.0 [18.0–20.2]	20.0 [19.0–20.0]	19.0 [17.5–20.5]	18.0 [16.8–20.0]	17.0 [16.0–21.0]	0.272
MR Severity						
None	13 (10.4%)	12 (22.6%)	0 (0%)	1 (9.1%)	0 (0%)	—
Mild	51 (40.8%)	41 (77.4%)	0 (0%)	10 (90.9%)	0 (0%)	—
Moderate	32 (25.6%)	0 (0%)	18 (54.5%)	0 (0%)	14 (50.0%)	—
Severe	29 (23.2%)	0 (0%)	15 (45.5%)	0 (0%)	14 (50.0%)	—
TR Severity						
None	20 (16.8%)	15 (31.2%)	5 (15.6%)	0 (0%)	0 (0%)	—
Mild	60 (50.4%)	33 (68.8%)	27 (84.4%)	0 (0%)	0 (0%)	—
Moderate	30 (25.2%)	0 (0%)	0 (0%)	9 (81.8%)	21 (75.0%)	—
Severe	9 (7.6%)	0 (0%)	0 (0%)	2 (18.2%)	7 (25.0%)	—

Data are presented as median [interquartile range] or n (%). LA = left atrial; LV = left ventricular; LVEF = left ventricular ejection fraction; MR = mitral regurgitation; RA = right atrial; RV = right ventricular; sPAP = systolic pulmonary artery pressure; TAPSE = tricuspid annular plane systolic excursion; TR = tricuspid regurgitation; Vmax = maximal velocity.

**Table 3:** Three-Dimensional Mitral Valve Parameters.

Parameter	No MR/TR (n = 53)	Isolated MR (n = 33)	Isolated TR (n = 11)	Combined MR + TR (n = 28)	*p*-Value
Annular perimeter, mm	121.0 [114.0–128.0]	130.0 [124.0–132.0]	124.0 [107.0–133.0]	129.0 [120.8–134.2]	0.009
Annular area, cm^2^	11.1 [10.0–12.7]	12.8 [11.6–13.6]	12.3 [8.9–13.9]	12.7 [11.2–13.9]	0.014
Anteroposterior diameter, mm	36.0 [32.0–39.0]	39.0 [36.0–41.0]	38.0 [33.0–40.5]	38.0 [36.0–41.2]	0.034
Commissural diameter, mm	36.0 [33.0–38.0]	37.0 [34.0–38.0]	38.0 [30.0–38.5]	37.0 [35.0–40.2]	0.274
Annular height, mm	6.9 [5.6–8.3]	8.1 [6.3–9.1]	4.6 [3.1–7.4]	7.8 [6.8–8.6]	0.019
Anterior leaflet length, mm	24.0 [21.0–26.0]	28.0 [27.0–29.0]	25.0 [21.5–27.0]	26.5 [24.0–31.0]	0.001
Posterior leaflet length, mm	15.0 [13.0–18.0]	15.0 [12.0–18.0]	14.0 [12.5–16.5]	16.0 [14.0–18.2]	0.268
Tenting height, mm	6.0 [4.0–8.0]	6.0 [5.0–8.0]	3.0 [3.0–6.0]	7.0 [6.0–9.0]	0.005
Tenting area, cm^2^	1.4 [1.1–1.9]	1.8 [1.4–2.2]	1.2 [0.8–1.7]	2.1 [1.5–2.6]	0.002

Data are presented as median [interquartile range]. *p*-values were calculated using the Kruskal–Wallis test across the four phenotypic groups. MR = mitral regurgitation; TR = tricuspid regurgitation.

**Table 4:** Factors associated with Combined Mitral and Tricuspid Regurgitation.

Variable	Univariable OR	Univariable 95% CI	Univariable *p*-Value	Multivariable OR	Multivariable 95% CI	Multivariable *p*-Value
Age, per 1 SD	1.49	0.92–2.40	0.103	1.18	0.65–2.12	0.584
Female sex	1.98	0.84–4.64	0.116	4.60	1.35–15.69	0.015
LVEF, per 1 SD	0.50	0.33–0.77	0.002	0.47	0.27–0.79	0.005
LA diameter, per 1 SD	1.76	1.11–2.80	0.017	1.78	0.99–3.21	0.054
RA diameter, per 1 SD	1.93	1.22–3.06	0.005	1.85	1.04–3.30	0.037
sPAP, per 1 SD	1.63	0.89–2.98	0.116	—	—	—
TAPSE, per 1 SD	0.52	0.22–1.23	0.138	—	—	—
RV basal diameter, per 1 SD	1.31	0.84–2.03	0.234	—	—	—
Beta-blocker	2.14	0.58–7.85	0.251	—	—	—
SGLT2 inhibitor	3.61	1.44–9.08	0.006	—	—	—
Loop diuretic	4.26	1.69–10.73	0.002	—	—	—

CI = confidence interval; LA = left atrial; LVEF = left ventricular ejection fraction; OR = odds ratio; RA = right atrial; RV = right ventricular; SD = standard deviation; SGLT2 = sodium-glucose cotransporter 2; sPAP = systolic pulmonary artery pressure; TAPSE = tricuspid annular plane systolic excursion. Model performance: AUC 0.81 (95% CI 0.71–0.91); Brier score 0.133; calibration slope 1.00; calibration intercept 0.00.

## Data Availability

The raw data supporting the conclusions of this article will be made available by the authors upon reasonable request and following approval by the University of Medicine and Pharmacy of Craiova, Romania. The data presented in this study are not publicly available due to privacy and ethical restrictions.

## Abbreviations

The following abbreviations are used in this manuscript:

AF	Atrial fibrillation
AFMR	Atrial functional mitral regurgitation
AFTR	Atrial functional tricuspid regurgitation
AUC	Area under the curve
CI	Confidence interval
EHRA	European Heart Rhythm Association
eGFR	Estimated glomerular filtration rate
IQR	Interquartile range
LA	Left atrium
LV	Left ventricle
LVEF	Left ventricular ejection fraction
MR	Mitral regurgitation
OR	Odds ratio
RA	Right atrium
RV	Right ventricle
SD	Standard deviation
SGLT2	Sodium-glucose cotransporter2
sPAP	Systolic pulmonary artery pressure
TAPSE	Tricuspid annular plane systolic excursion
TEE	Transesophageal echocardiography
TR	Tricuspid regurgitation
